# Ovarian cancer detection by DNA methylation in cervical scrapings

**DOI:** 10.1186/s13148-019-0773-3

**Published:** 2019-11-27

**Authors:** Tzu-I Wu, Rui-Lan Huang, Po-Hsuan Su, Shih-Peng Mao, Chen-Hsuan Wu, Hung-Cheng Lai

**Affiliations:** 1 Department of Obstetrics and Gynecology, School of Medicine, College of Medicine, Taipei Medical University, Taipei, Taiwan; 2 Department of Obstetrics and Gynecology, Wan Fang Hospital, Taipei Medical University, Taipei, Taiwan; 3 Department of Obstetrics and Gynecology, Shuang Ho Hospital, Taipei Medical University, New Taipei, Taiwan; 4 Translational Epigenetic Center, Shuang Ho Hospital, Taipei Medical University, New Taipei, Taiwan; 5 Graduate Institute of Clinical Medical Sciences, Chang Gung University College of Medicine, Tao-Yuan, Taiwan; 6 Department of Obstetrics and Gynecology, Kaohsiung Chang Gung Memorial Hospital and Chang Gung University College of Medicine, Kaohsiung, Taiwan; 7 Department and Graduate Institute of Biochemistry, National Defense Medical Center, No.291, Jhongjheng Rd., Jhonghe, New Taipei, 23561 Taiwan

**Keywords:** Cancer detection, Ovarian cancer, DNA methylation, Cervical scrapings

## Abstract

**Background:**

Ovarian cancer (OC) is the most lethal gynecological cancer, worldwide, largely due to its vague and nonspecific early stage symptoms, resulting in most tumors being found at advanced stages. Moreover, due to its relative rarity, there are currently no satisfactory methods for OC screening, which remains a controversial and cost-prohibitive issue. Here, we demonstrate that Papanicolaou test (Pap test) cervical scrapings, instead of blood, can reveal genetic/epigenetic information for OC detection, using specific and sensitive DNA methylation biomarkers.

**Results:**

We analyzed the methylomes of tissues (50 OC tissues versus 6 normal ovarian epithelia) and cervical scrapings (5 OC patients versus 10 normal controls), and integrated public methylomic datasets, including 79 OC tissues and 6 normal tubal epithelia. Differentially methylated genes were further classified by unsupervised hierarchical clustering, and each candidate biomarker gene was verified in both OC tissues and cervical scrapings by either quantitative methylation-specific polymerase chain reaction (qMSP) or bisulfite pyrosequencing. A risk-score by logistic regression was generated for clinical application.

One hundred fifty-one genes were classified into four clusters, and nine candidate hypermethylated genes from these four clusters were selected. Among these, four genes fulfilled our selection criteria and were validated in training and testing set, respectively. The OC detection accuracy was demonstrated by area under the receiver operating characteristic curves (AUCs) in 0.80–0.83 of *AMPD3*, 0.79–0.85 of *AOX1*, 0.78–0.88 of *NRN1*, and 0.82–0.85 of *TBX15*. From this, we found OC-risk score, equation generated by logistic regression in training set and validated an OC-associated panel comprising *AMPD3*, *NRN1*, and *TBX15*, reaching a sensitivity of 81%, specificity of 84%, and OC detection accuracy of 0.91 (95% CI, 0.82–1) in testing set.

**Conclusions:**

Ovarian cancer detection from cervical scrapings is feasible, using particularly promising epigenetic biomarkers such as *AMPD3/NRN1/TBX15*. Further validation is warranted.

## Background

Ovarian cancer (OC) is the fifth-leading cause of cancer death in the USA, and the most lethal female genital tract malignancy worldwide, with over 150,000 deaths in 2012 [1]. Important compelling reasons for its lethality are its vague and nonspecific symptoms that are often disregarded in early stage disease, when overall survival (OS) is 86–93%. By contrast, the more uncomfortable abdominal pain, fullness, or annoying gastrointestinal problems are often not noticed until the disease reaches stage III/IV status, comprising the majority (> 75%) of women with OC. Consequently, although for localized OC, the overall survival (OS) is 86–93%, only 25% of all diagnostic presentations occur at this time, and the OS drops to 21–30% for advanced stage cases [2, 3].

With regard to therapies, while treatment advances have boosted survival outcomes for many types of cancer, over the past two decades, OC has seen slower progress. Thus, despite successful efforts in improving OC treatment, including surgery, cytotoxic chemotherapy, hyperthermic intraperitoneal chemotherapy, and targeted therapy, only marginal improvement has been seen [4, 5]. Therefore, while feasible, effective early screening/detection strategy for OC is of utmost urgency, recent aggressive attempts at developing early detection approaches, using traditional imaging and serum biomarkers, have failed to reduce morbidity and mortality [6].

One much-studied, potential early detection approach, the use of the serum biomarker cancer antigen 125 (CA-125) and transvaginal ultrasound (TVU), was extensively examined in the Prostate, Lung, Colorectal, and Ovarian (PLCO) cancer screening trial, including 78,216 women, with a median follow-up up to 13 years. That study showed no mortality benefit across an OC screening and no screening arm. This diagnostic evaluation also yielded a high false-positive rate associated with surgical complications [3]. Another large OC screening trial, the UK Collaborative Trial of Ovarian Cancer Screening (UKCTOCS), observed more than 200,000 women, with a median follow-up of 11 years, revealing no significant reduction of mortality in the primary analysis. However, the early-stage shift was demonstrated as 37.8%, 23%, and 24% in the annual multimodal screening (MMS) by serum CA-125 interpreted with use of the risk of ovarian cancer algorithm, annual TVU, and no screening groups, respectively, in UKCTOCS trial. Long-term follow-up is needed before firm conclusion is reached on the efficacy and cost-effectiveness of OC screen [7]. Thus to date, no clinical practice guideline has supported current OC screening tools, including TVU and CA-125, for the early detection of OC.

To augment (i.e., decrease false positive) traditional screening tools (TVU and CA-125), novel molecular biomarkers are now under intense study. To that end, the inclusion of additional blood-based protein biomarkers, such as HE4 or CA72-4, was found encouraging [8–10]. Even so, the results have not yet proven sufficiently sensitive or reproducible to be used clinically. Liquid biopsies, which detect circulating tumor cells (CTCs) or circulating tumor DNA (ctDNA), from blood, have also been promising, although current results have not supported their general use for OC screening [11–15], and their prospective evaluation (i.e., clinical trials) remains lacking [16, 17]. Because the aforementioned studies have included mainly late-stage patients, the utility of these methods for detecting early-stage disease is uncertain.

For the detection of cervical cancer, the Papanicolaou (Pap) test collects endocervical samples, although ovarian and endometrial cancers (ECs) are infrequently detected via abnormal cervical cytology. Recently, one study demonstrated that DNA mutational analysis of Pap samples was capable of detecting OCs and ECs. In that work, massive parallel sequencing of 12 exons of *APC*, *AKT1*, *BRAF*, *CTNNB1*, *EGFR*, *FBXW7*, *KRAS*, *NRAS*, *PIK3CA*, *PPP2R1A*, *PTEN*, and *TP53*, from Pap test specimens, was able to identify 41% of OCs (9 of 22), potentially opening OC detection to a new panel of molecular biomarkers found in cervical Pap smears [18].

In addition to genetic events, epigenetic changes have been widely studied in cancer. For example, DNA hypermethylation-mediated silencing of tumor suppressor genes is common in overall carcinogenesis, such that research regarding epigenetic alterations in OC have also been associated with different histologies, grades, stages, response to chemotherapy or targeted therapy, relapse risk, and survival [19–21]. Our previous proof-of-concept study also demonstrated the possibility of OC detection by DNA methylation analysis of cervical scrapings [22], prompting us here to more thoroughly investigate OC-specific DNA methylation biomarkers in conventional Pap test, including exploration of their clinical performance.

## Results

### Differential methylation analysis of ovarian cancer tissues and cervical scrapings

**Fig. 1:**
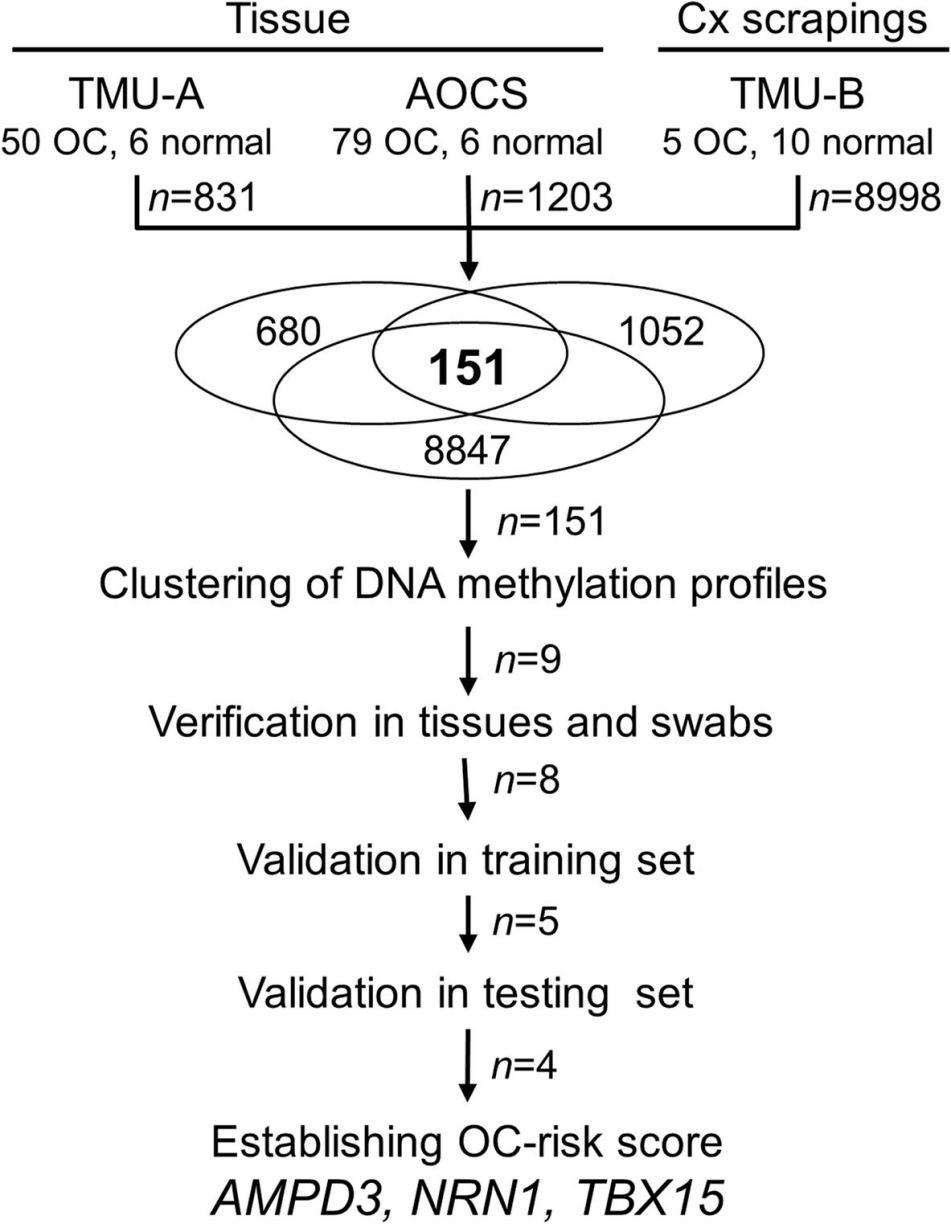
Definition of differentially hypermethylated genes of serous ovarian carcinoma patients. Flowchart for discovering candidate genes, and the intersection of three methylomics datasets to distinguish ovarian carcinomas, from normal controls, in cervical scrapings. OC, ovarian carcinoma; TMU-A, Taipei Medical University-A ovarian tissue dataset. AOCS, the Australian Ovarian Cancer Study ovarian tissue dataset. TMU-B, Taipei Medical University-B cervical scraping dataset

The logistics of the present study is illustrated in Fig. 1. The methylomics profiles from Taipei Medical University-A (TMU-A) ovarian tissue dataset, Australian Ovarian Cancer Study (AOCS)–ovarian tissue dataset and TMU-B cervical scraping dataset were used to identify highly differentially methylated (HDM) genes between serous OC and non-OC patients. These selected HDM genes belonged to the intersection of all statistically significantly hypermethylated genes shown in these three datasets. The detailed clinicopathological features of these three datasets are described in Additional file 1: Table S1, and older age OC patients in the TMU-A (mean age ± standard deviation: 58.1 ± 12.1 vs. 51.3 ± 16.4 years) and TMU-B (65.8 ± 14.0 vs. 40.9 ± 4.8 years) were noticed when compared with normal controls. Stage I/II cases accounted for 22% and 40% in the TMU-A and TMU-B datasets, respectively, but no early stage samples were found in the ACOS dataset. The distribution of grading also showed that among the three datasets’ methylomics profiles, 831 and 1203 HDM genes were found in the TMU-A and AOCS ovarian cancer tissues datasets, respectively, as well as 8998 HDM genes in the TMU-B cervical scrapings dataset. The intersection of all HDM genes from these three datasets revealed 151 genes (Fig. 1, Additional file 1: Figure S1 and Table S4). Bioinformatics analysis of these 151 HDM genes using the Database for Annotation, Visualization and Integrated Discovery (DAVID, version 6.8), Kyoto Encyclopedia of Genes and Genomes (KEGG, http://www.kegg.jp/ or http://www.genome.jp/kegg/) and Reactome pathway databases showed enrichment in several signaling pathways, including maturity-onset diabetes of the young, peptide ligand-binding receptors, and the estrogen signaling pathway (Additional file 1: Table S2).

### Methylation clustering of ovarian cancer

**Fig. 2:**
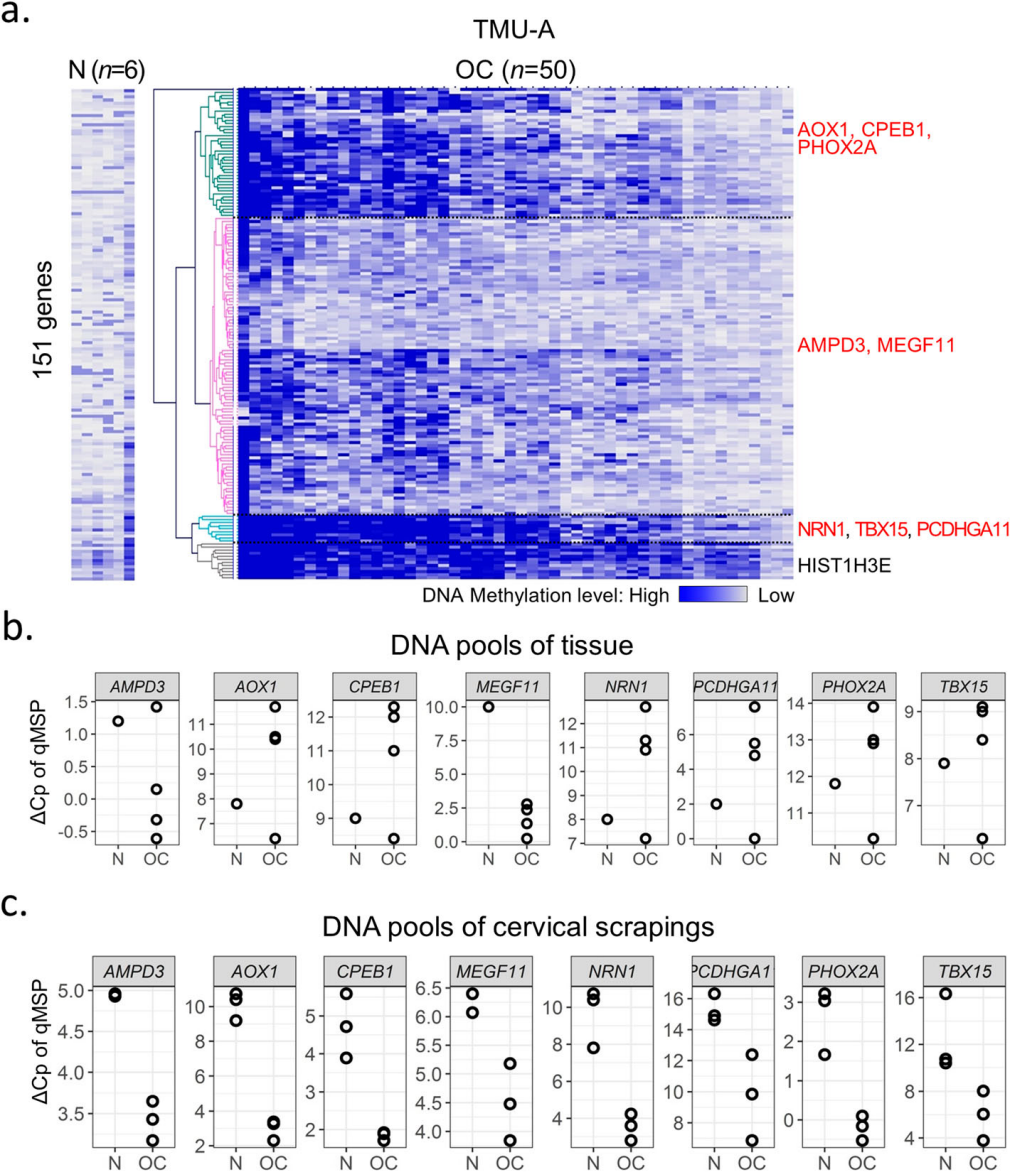
Selection and verification of candidate genes. **a** Hierarchical clustering analysis of potential candidate genes with methylation profiles. The heatmap represents DNA methylation levels and clustering into 4 subgroups. We verified the top 10% of hypermethylated genes, in each group. If more than 5 hypermethylated genes were shown, we chose 2 or 3 genes of each subgroup and less reported in literature which listed on the right side. **b** and **c** DNA methylation levels of candidate genes were verified by quantitative methylation-specific PCR (qMSP), using DNA pooled from tissues and cervical scrapings. Each dot shows 5 specimens with the same diagnosis in a pooled DNA. TMU-A, Taipei Medical University-A ovarian tissue dataset; N, normal; OC, ovarian carcinoma

We utilized these 151 HDM genes which were listed in detail (Additional file 1: Table S4) to conduct unsupervised hierarchical clustering analysis for candidate gene selection, showing clustering of four subgroups (Fig. 2a). We selected top 10% of HDM genes in each clustering subgroup (Fig. 2a). Those less reported in the literature were set as the priority, which narrowed down to a list of nine genes. Nine candidate genes underwent further testing by either quantitative methylation-specific polymerase chain reaction (qMSP) or bisulfite pyrosequencing, including *AOX1*, *CPEB1*, *PHOX2A*, *AMPD3*, *MEGF11*, *NRN1*, *TBX15*, *PCDHGA11*, and *HIST1H3E* (Additional file 1: Table S2, S5*)*.

### Verification of highly differentially methylated genes

Of the aforementioned nine genes, eight were successfully verified by qMSP assays, and one gene, *HIST1H3E*, by bisulfite pyrosequencing, in DNA pools of either tissues or cervical scrapings (Fig. 2b, c and Additional file 1: Figure S2). Genes with a qMSP cycle difference of crossing points (ΔCp) from OCs lower than those from the normal controls, in at least one DNA pool of OC tissues, and in all three DNA pools of cervical scrapings, were selected for further testing. To keep representative for most patients, we selected 1–2 candidates with the highest value of ΔCp from each clustering subgroup (Fig. 2a, Additional file 1: Table S4). The qMSP condition of *CPEB1* is unstable in the following testing of individual samples. Therefore, we excluded the gene in the following analysis. *HIST1H3E* from the subgroup 4 was verified successfully by bisulfite pyrosequencing, but not by qMSP due to primer issues. Therefore, a final count of five genes, *AMPD3*, *AOX1*, *MEGF11*, *NRN1*, and *TBX15*, passed all these criteria and selected from the three clustering subgroups (Fig. 2b, c). The detailed value of ΔCp, for each candidate gene, and related clustering subgroups are described in Additional file 1: Table S5.

### Validation of DNA methylation by training and testing sets in cervical scrapings

**Fig. 3:**
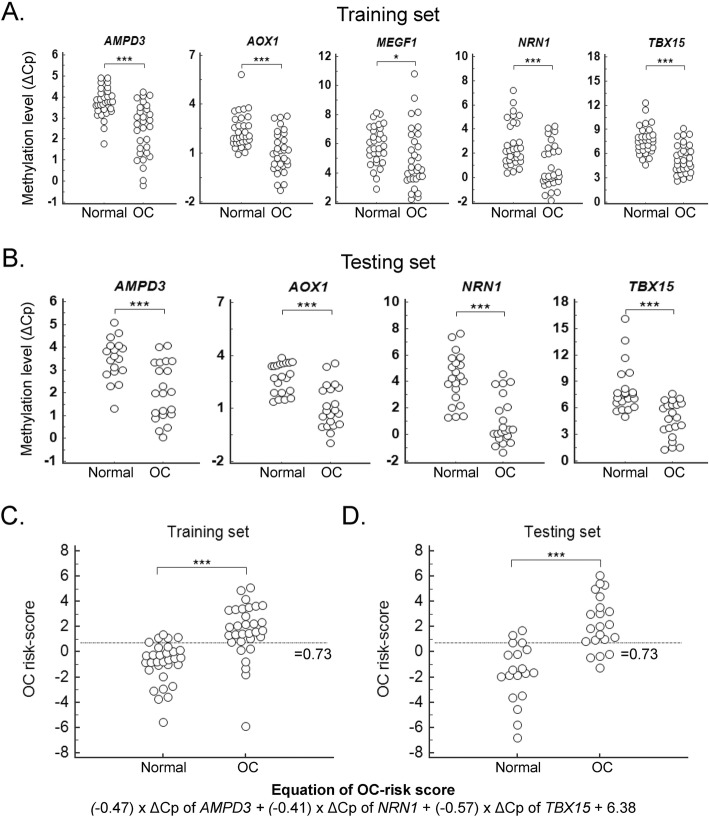
Validation of DNA methylation levels in training and testing sets, and construction of OC-risk scores. **a** and **b** Distribution of DNA methylation levels in cervical scrapings from training and testing sets. We detected the methylation levels of *AMPD3*, *AOX1*, *NRN1*, and *TBX5*, and used those with the better significance for distinguishing normal controls and ovarian carcinomas, in the training set. These four genes also confirmed a significant difference between normal controls and ovarian carcinomas in the testing set. The distribution of risk score in cervical scrapings from the training set (**c**) and testing set (**d**). *P* values were compared with normal and disease using two-tailed Mann–Whitney *U* test. ***< 0.001; *< 0.05. OC-risk score equation = (− 0.47) × ΔCp of *AMPD3* + (− 0.41) × ΔCp of *NRN1* + (− 0.57) × ΔCp of *TBX15* + 6.38. OC, ovarian carcinoma; AUC, area under the receiver operating characteristic curve; Sen., sensitivity; Spe., specificity

The clinicopathological features of the OC patients in the training and testing sets are shown in Table 1. We then quantified methylation levels of these candidate genes, in both training and testing sets (Table 2). All five genes, *AMPD3*, *AOX1*, *MEGF11*, *NRN1*, and *TBX15*, were statistically significantly hypermethylated in cervical scrapings from OC patients in the training set, and four of five genes with area under the receiver operating characteristic curves (AUCs) greater than 0.7, except *MEGF11*, were subject to further validation in the testing set. The distribution of the depicted plots represents the methylation levels, in terms of change in PCR threshold cycle (ΔCp value) of each candidate gene, between normal and OC cervical scrapings in the training and testing sets, respectively. The results all reached statistically significant differences (Fig. 3a, b). The corresponding cut-off values ofΔCp, sensitivity, specificity, and AUC of each candidate gene, or genetic combination, are listed, for both the training and testing sets (Table 3). 57–76% sensitivity and 71–100% specificity, and 0.83–0.88 AUC were validated using single genes in the testing set. Combinations improved the accuracy; in particular, the combination of *AMPD3*, *NRN1*, and *TBX15* conferred the best accuracy, with an AUC of 0.91 (95% CI, 0.82–1) (Table 3).

**Table 1:** Clinicopathological features of cervical scrapings in training and testing set

		Training set		Testing set		*P* value
		OC	Normal	OC	Normal	
Total number		31	31	21	21	
Age (years)	Mean ± SD	52.7 ± 13.4	45.5 ± 12.9	51.4 ± 11.9	40.8 ± 13.0	
FIGO stage	Stage 1	13 (41.9%)		6 (28.6%)		0.23
	Stage 2	5 (16.1%)		2 (9.5%)		
	Stage 3	9 (29%)		12 (57.1%)		
	Stage 4	4 (12.9%)		1 (4.8%)		
Grading	G1	4 (12.9%)		3 (14.3%)		0.66
	G2	6 (19.4%)		2 (9.5%)		
	G3	19 (61.3%)		13 (61.9%)		
	Unknown	2 (6.5%)		3 (14.3%)		
Histology	Ser	16 (51.6%)		14 (66.7%)		0.25
	En	3 (9.7%)		0 (0%)		
	Mu	7 (22.6%)		2 (9.5%)		
	CC	5 (16.1%)		5 (23.8%)		

*OC*, ovarian carcinoma; *SD*, standard deviation; FIGO stage, it followed the International Federation of Gynecology and Obstetrics staging system to identify the stage. *Ser*, serous; *En*, endometrioid; *Mu*, mucinous; *CC*, clear cell

**Table 2:** Summary DNA methylation level of candidate genes in training and testing sets

Sample set	Diagnosis	No.	AMPD3	AOX1	MEGF1	NRN1	TBX15
Training set ΔCp median ± (95% CI)	Normal	31	3.8 ± (3.5–4.0)	2.0 ± (1.8–2.7)	5.8 ± (5.3–6.5)	2.3 ± (1.6–2.9)	7.6 ± (6.8–8.1)
	OC	31	2.7 ± (1.8–3.2)***	1.0 ± (0.3–1.5)* **	4.4 ± (3.8–5.7)*	0.1 ± (−0.1 − 1.9)***	5.1 ± (4.2–6.2)***
Testing set ΔCp median ± (95% CI)	Normal	21	3.6 ± (3.0–4.1)^a^	2.8 ± (2.0–3.5)	–	4.2 ± (3.2–5.3)	7.6 ± (6.7–8.0)
	OC	21	2.0 ± (1.1–3.1)***	0.9 ± (0.2–2.0)* **	–	0.4 ± (− 0.0 − 1.9)***	4.9 ± (3.7–6.3)***

*No.*, number of cases; *CI*, confidence interval; *OC*, ovarian carcinoma

*P* values were compared with normal and disease using two-tailed Mann–Whitney *U* test. ***< 0.001; *< 0.05

^a^The number of qualified values is 19

**Table 3:** The DNA methylation of cervical scrapings in discriminating normal and ovarian carcinoma patients

Gene set	Training set				Testing set		
	Cut-off	Se. (%)	Sp. (%)	AUC (95% CI)	Se.(%, 95% CI)	Sp. (%, 95% CI)	AUC (95% CI)
Single gene							
*AMPD3*	3.10	64.5	90.3	0.80 (0.68–0.91)	71.4 (47.8–88.7)	71.4 (47.8–88.7)	0.83 (0.70–0.95)
*AOX1*	1.29	64.5	93.6	0.79 (0.67–0.91)	66.7 (43.0–85.4)	100 (83.9–100)	0.85 (0.73–0.97)
*MEGF1*	4.47	54.8	87.1	0.68 (0.54–0.82)	–	–	–
*NRN1*	0.46	61.3	96.8	0.78 (0.67–0.90)	57.1 (34.0–78.2)	100 (83.9–100)	0.88 (0.79–0.98)
*TBX15*	6.37	74.3	83.9	0.82 (0.71–0.92)	76.2 (52.8–91.8)	76.2 (52.8–91. 8)	0.85 (0.74–0.97)
Gene combination^a^							
*AMPD3 + TBX15*	− 0.30	87.1	74.2	0.85 (0.75–0.95)	90.5 (69.6–98.8)	52.4 (29.8–74.3)	0.88 (0.79–0.98)
*AOX1 + TBX15*	0.85	74.2	90.3	0.85 (0.74–0.95)	71.4 (47.8–88.7)	85.7 (63.66–97.0)	0.88 (0.79–0.98)
*NRN1 + TBX15*	1.92	77.4	83.9	0.85 (0.75–0.95)	57.1 (34.0–78.2)	95.2 (76.2–99.98)	0.90 (0.81–0.99)
*AMPD3 + NRN1 + TBX15*	0.73	80.7	83.9	0.87 (0.77–0.97)	81.0 (58.1–94.6)	84.2 (60.4–96.9)	0.91 (0.82–1.0)
*AOX1 + NRN1 + TBX15*	1.93	77.4	83.9	0.85 (0.75–0.95)	57.1 (34.0–78.2)	95.2 (76.2–99.9)	0.90 (0.81–0.99)
*AOX1 + AMPD3 + TBX15*	0.37	77.4	87.1	0.85 (0.75–0.96)	57.1 (34.0–78.2)	94.7 (74.0–99.9)	0.89 (0.80–0.99)

*Se*., sensitivity; *Sp*. Specificity; *AUC*, area under the curve of receiver operating characteristic; *CI*, confidence interval; *OC*, ovarian carcinoma

^a^The accuracy of gene combinations was estimated by the logistics regression model

### Clinical performance of an integrated model to predict risk of ovarian cancer

**Fig. 4:**
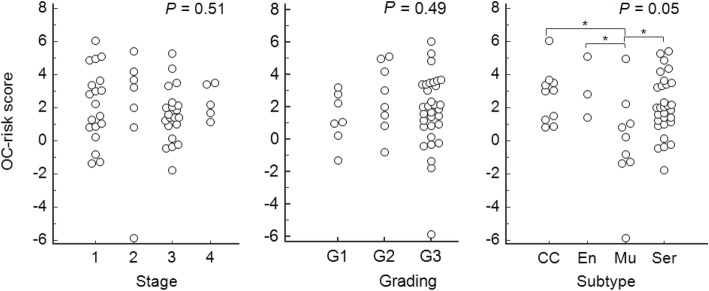
The distribution of OC-risk score in stage, grading and subtypes from cervical scrapings of ovarian cancer patients. The methylation level of OC showed no difference in stages and grading. However, the methylation level of mucinous OC showed significant lower than other histological types. *P* values were calculated by Kruskal–Wallis test. *Showed the post hoc test < 0.05. OC, ovarian carcinoma; CC, clear cell; En, endometrioid; Mu, mucinous; Ser, serous

To translate the results of our findings for clinical application, we developed a mathematical equation for risk prediction of OC (OC-risk score), by integrating methylation levels of *AMPD3*, *NRN1*, and *TBX15*. A logistic regression model including 62 cervical scrapings from training set was used to formulate a robust OC-risk score model (Fig. 3c). A cut-off value of 0.73 generated by an equation of (− 0.47) × ΔCp of *AMPD3 +* (− 0.41) × ΔCp of *NRN1* + (− 0.57) × ΔCp of *TBX15* + 6.38 resulted in a sensitivity of 80.7% and a specificity of 83.9%. Then, the cut-off value, 0.73, was applied to 42 cervical scrapings from testing set (Fig. 3d). The sensitivity and specificity was 81.0% and 84.2%, respectively. The correlation of OC-risk score to clinical parameters was tested. The differences among different histology types were statistically significant (*P* < 0.05). Mucinous type has a lower OC-risk score (Fig. 4). We analyzed the association between age and methylation levels of candidate genes for the concern of age effect. The results showed non-significant association (all *P* values > 0.05) and listed in Additional file 1: Table S6.

## Discussion

Only 25% of high-grade serous ovarian cancers are only diagnosed in early stages, underscoring an urgent need for practical means of screening. Prior large-scale efforts have assessed the efficacy of OC screening, using different modalities such as serum CA-125 levels and transvaginal ultrasound imaging, including the Prostate, Lung, Colorectal, and Ovarian Cancer (PULCO) [3] and UK Collaborative Trial of Ovarian Cancer Screening (UKCTOCS) [7] trials. However, these screening trials did not show improved mortality, to date, but rather, increased false positive rates and related surgical complication [6, 23]. Moreover, the value of general OC screening in the postmenopausal female population remains controversial, and one perspective is that to date, it may actually do more harm than good [6]. Here, we discovered ovarian cancer (OC)–specific hypermethylated genes. Hopefully, the emergence of novel molecular markers could change the debate toward a willingness for further development of OC screening.

Recently, the use of serum proteins (CA-125, CA-199, CEA, prolactin, hepatocyte growth factor, osteopontin, myeloperoxidase, and tissue inhibitor of metalloproteinases-1) in combination with 13 cell-free (cf)-DNA amplicons (*NRAS*, *CTNNB1*, *PIK3CA*, *FBXW7*, *APC*, *EGFR*, *BRAF*, *CDKN2A*, *PTEN*, *FGFR2*, *HRAS*, *AKT1*, *TP53*), i.e., the “CancerSEEK” blood test, was reported to detect multiple cancers, including OC [24, 25]. While the sensitivities of OC detection reached 98%, there were only 54 OC patients in that study, and most of them were in late stages (77.8%) [25].

The introduction and widespread uptake of regular cervical screening with the Pap test or cervical scrapings, is the main cause of reduced incidence, and associated deaths from cervical cancer (CC). To simultaneously utilize such easily accessing approaches (e.g., Pap test/cervical scrapings), for the discovery of OC detection biomarkers, is appealing. One study even illustrated that DNA mutational analyses of samples collected from cervical scrapings could detect ovarian and endometrial cancer [18]. Due to sensitive massively parallel sequencing, OC can be detected, although the detection rate remained low (41%, 9 of 22). Thus, cervical scrapings could be even more advantageous for the detection of diseases of the internal female genital tract. “Sloughed-off” cancer cells, and cellular fragments, into the endocervical canal are considered the most likely mechanisms for the appearance of such anomalous cells. Indeed, although rare, some OC cells can be identified by conventional cytology in Pap tests [26, 27]. Thus, Pap testing for OC detection may be improved if novel molecular markers are discovered.

Recently, one study using a Pap brush, called PapSEEK detected 18 genetic mutations, including *AKT1*, *APC*, *BRAF*, *CDKN2A*, *CTNNB1*, *EGFR*, *FBXW7*, *FGFR2*, *KRAS*, *MAPK1*, *NRAS*, *PIK3CA*, *PIK3R1*, *POLE*, *PPP2R1A*, *PTEN*, *RNF43*, and *TP53*, in addition to revealing chromosomally aneuploid OC cells, at a detection sensitivity of 33% [28]. If, in place of cervical smears, PapSEEK obtained tissue material from the relatively invasive intrauterine Tao brush or lavage, the sensitivity of this approach could reach 45% [28]. Our previous proof-of-concept study demonstrated the possibility of OC detection by testing hypermethylation of *PTGDR*, *HS3ST2*, *POU4F3*, and *MAGI2* genes from cervical scrapings [22]. However, these genes were discovered from cervical cancer dataset, which were not included in the candidate list using OC dataset. The present study discovered OC-specific hypermethylated genes demonstrated a sensitivity of 61–76%, and an accuracy of 0.78–0.88 to detect OC by single candidate genes. Furthermore, the combinations of *AMPD3*, *NRN1*, and *TBX15* discovered increased sensitivity of 81%, and increased accuracy of 0.87–0.91.

The functional role of these genes in OC remains unexplored. *AMPD3* (adenosine monophosphate deaminase 3) encodes a member of the adenosine monophosphate (AMP) deaminase gene family, and its encoded protein belongs to a highly regulated enzyme that catalyzes the hydrolytic deamination of AMP to inosine monophosphate (IMP), in the adenylate cyclase catabolic pathway [29]. *AOX1* (aldehyde oxidase 1) produces hydrogen peroxide, and can catalyze the formation of superoxide, under certain conditions. Much less is known about the physiological function of the enzymatic substrates/products of human AOX1, and other mammalian AOX isoenzymes [30]. One of these, *NRN1* (Neuritin 1), encodes a member of the neuritin family, which is expressed in postmitotic-differentiating neurons of the developmental nervous system. *NRN1* participates in promoting migration of neuronal cells, and impacts microtubule stability [31]. Another one, *TBX15* (T-box-15), belongs to the T-box family of genes, which encode a phylogenetically conserved family of transcription factors that regulate a variety of developmental processes [32]. None of these genes has been reported in OC.

The combination of three candidate genes, *AMPD3*, *NRN1*, and *TBX15*, reached the detection accuracy as 0.87–0.91 of AUC to distinguish OCs from normal controls in our current study. Although these selected genes retrieved from the three methylomics datasets containing serous OCs specifically, the detection accuracy in varied histological type of OCs might be different but remained promising. The different distribution of OC-risk score between mucinous and non-mucinous OCs was observed significantly, and the difference of OC-risk score in different histology types is interesting. Different origins or different tumor behaviors may cause the difference of methylation profiles in tumors and in cervical scrapings [33]. The possible speculation is that the precursors of mucinous OCs from the gastrointestinal tract obviously differ from precursors of non-mucinous OCs from Müllerian duct during embryological development. Further clarification of ovarian cancer type-specific methylation in cervical scrapings is warranted.

Although promising, our study has several limitations. First, it is a discovery phase from a retrospective case-control study. The results here are not yet appropriate for dissemination to the general population. Second, confounding by other uterine or ovarian neoplasms, or disrupting anatomical location remains to be determined. According to our previous studies and literature [22], different cancers may have common gene methylations. Whether *AMPD3/NRN1/TBX15* methylations may occur in other gynecological cancers or in benign tumors remains to be determined. The epigenetic alteration influenced by hormone, infection, inflammation or oxidative stress factors remains doubtful in the detection accuracy as well as the issue of disrupting conduit of cellular debris from ovary/fallopian tube into endocervical canal (i.e., tubal sterilization, intrauterine device insertion, salpingectomy or supracervical hysterectomy). Third, epithelial OCs themselves are heterogeneous in histology types, with different etiologies. It raises challenges that epithelial OCs comprise of a large heterogeneity dividing into different subtypes according to their morphological, clinical, and molecular genetic characteristics. To solve these limitations before clinical application, further validation in population-based prospective clinical trial is warranted.

## Conclusion

The potential development of DNA methylation biomarkers, from cervical scrapings, expands the scope of the Pap test, a now-routinely used cytological exam especially prevalent in developed countries. The detection of female genital tract malignancies, including CC, EC, and OC, by combining cervical scrapings and molecular markers, is an attractive concept. Here, we revealed DNA methylation of the genes *AMPD3*, *NRN1*, and *TBX15* as promising biomarkers for OC detection. Further, large-scale trials are needed to validate the potential of these procedures and the use of such promising biomarkers.

## Methods

### Study design and clinical samples

We enrolled a total 205 participants, aged 20 to 90 years old, and collected 149 cervical scrapings and 50 malignant and 6 normal epithelial ovarian tissues. Participants signed informed consent for the study, between November 2014 and October 2017, at Shuang Ho Hospital and Wan Fang Hospital, Taipei Medical University, Taipei, Taiwan. The study was conducted strictly according to a protocol approved by the Institutional Review Board of the Taipei Medical University, in accordance with the Declaration of Helsinki, 2000. Cervical scrapings were obtained in operation room or during an outpatient visit before initial surgery, using a cervical brush (60011 LIBO Conical nylon brush, Iron Will Biomedical Technology, New Taipei, Taiwan). Normal ovarian epithelial cells were obtained from participants diagnosed with uterine leiomyomas, after abdominal total hysterectomy combined with salpingo-oophorectomy. All specimens were collected and placed immediately in RNA*later*® Stabilization Solution (ThermoFisher, Waltham, MA, USA). We then liquated the cervical scrapings after vortexing for 1 min, followed by storage at − 80 °C, until DNA extraction. Age, histological type of tumor, International Federation of Gynecology and Obstetrics (FIGO) stage, and histological grade were tabulated in the hospital records for each anonymized participant. Ovarian tissues (50 OCs vs. 6 normal controls) and cervical scrapings (5 OCs vs. 10 normal controls) were utilized for methylomics analysis, respectively. We randomly selected cervical scrapings from 15 OCs and 15 normal controls for verification. Every 5 cervical scrapings from OCs or normal controls were put together as one DNA pool and depicted as one dot in Fig. 2c. The remaining 104 cervical scrapings were used for validation, including 31 OCs plus 31 normal controls from training set and 21 OCs plus 21 normal from testing set in Table 1.

For validation, the samples size, estimated at AUC 0.75 for each candidate gene, compared with AUC 0.5 as the null hypothesis status, with 0.05 as the type I error (α), 0.2 as the type II error (β, 1-power), and a 1:1 ratio of OC case numbers to normal groups. Accordingly, we assigned a ratio of the sample size of training set at 1.5-fold that of the testing set. Two samples were added to both the OC and normal groups to avoid a failed detection. The sample sizes of the training and testing sets were predicted to be 62 and 42, respectively. We enrolled participants between November 2014 and August 2016 for the training set, and from August 2016 to October 2017 for the testing set. Clinicopathological results and demographics are listed in Table 1 and Additional file 1: Table S1.

### Differential methylomics and bioinformatics analysis

For identifying highly differentially methylated (HDM) OC genes, we generated two methylomics profiles for tissues and cervical scrapings, respectively, and one public dataset. Taipei Medical University set A (TMU-A) ovarian tissues were analyzed for DNA methylomics profiles, using pull-down by the methyl-CpG-binding domain protein 2 (MBD2), followed by high-throughput, next-generation sequencing [34]. We then calculated HDM regions between 50 serous-type OCs and 6 normal ovarian epithelia from TMU-A, using uniquely mapped reads, to represent DNA methylation levels. We specifically focused on the methylation level of a 2000-bp region spanning 1000 bp upstream and downstream of the transcriptional start site (TSS) of coding genes of interest (reference genome of UCSC version hg18), as annotated with NM-type (RNA) RefSeq accessions, and excluded coding genes on sex chromosomes. The methylation levels of all the sample genes were normalized to separated, total mapped reads. Significantly HDM genes were identified by Mann–Whitney *U* test with *P* < 0.01, HDM level > 0.2, and AUC > 0.85.

We also used another public methylome OC tissue dataset to assist discovery of potential OC-specific HDM biomarkers. The Australian Ovarian Cancer Study (AOCS)–tissue dataset was analyzed using the HumanMethylation450 BeadChip (Illumina, San Diego, CA, USA) and deposited in the NCBI’s Gene Expression Omnibus (GEO) with accession number GSE65820 [35]. In the bead-chip system, we used β-values to present DNA methylation level of each probe, which is remained by detecting *P* value ≤ 0.01, the number of single nucleotide polymorphism (SNP) ≥ 2, genes annotated with NM-type RefSeq accessions, and excluded genes coded on sex chromosomes. We analyzed HDM probes by comparison with 79 primary serous-type OCs and 6 normal fallopian tubes from AOCS dataset. The fallopian tube epithelia rather than ovarian surface epithelia have been considered to be the origin of high-grade serous OC according to the previous epidemiologic studies (i.e., BRCA mutation carriers underwent risk reducing salpingo-oophorectomy surgery), molecular genetic pathologic studies, and methylome analysis [36, 37]. Significant HDM genes were identified by including HDM levels for each probe > 0.15, Mann–Whitney *U* test with *P* < 0.05, AUC > 0.75, and the number of HDM probes at a promoter region of the closest gene ≥ 3.

To identify OC-specific HDM genes by cervical scrapings, we assayed the Taipei Medical University set B (TMU-B) cervical scrapings dataset to construct methylomics profiles of 5 OC and 10 healthy control cervical scrapings, using the HumanMethylation450 BeadChip. Each pooled DNA contained equal amounts of DNA from 5 specimens. HDM genes were identified by including HDM level of each probe > 0.015, and the number of probes at a promoter region of the closest gene ≥3.

For selecting candidate HDM genes, methylation profiles were grouped by unsupervised hierarchical clustering analysis, with complete-linkage and Euclidean distance methods performed using Multiple Experiment Viewer (MeV) version 4.9 (https://sourceforge.net/projects/mev-tm4/) [38]. One hundred fifty-one HDM genes represented the intersection of the three datasets (TMU-A, TMU-B, and AOCS), which were conducted using the TMU-A dataset for further hierarchical clustering analysis. When each subgroup comprised of more than five HDM genes, we selected the top 10% differential methylation levels, and less reported genes in the literature, for further investigation.

For better understanding of the biological effects of the 151 HDM genes, functional enrichment annotation was performed using public tools, the Database for Annotation, Visualization and Integrated Discovery DAVID (version 6.8) [39] and KEGG (http://www.kegg.jp/ or http://www.genome.jp/kegg/) [40]**,** and Reactome [41] pathway databases. A threshold of *P* ≤ 0.05 was used for enriched annotation (Additional file 1: Table S2).

### DNA preparation and methylation level detection

Genomic DNA was extracted from cervical scrapings and tissues using the QIAamp DNA Mini Kit (QIAGEN, Hilden, Germany), and its concentration detected using a Nanodrop 1000 (Thermo Fisher Scientific, Waltham, MA, USA). Pooled DNA contained DNA from five specimens. DNA was bisulfite-converted from 1-μg genomic DNA, using the EZ DNA Methylation Kit (Zymo Research Corp., Irvine, CA, USA), according to the manufacturer’s recommendations of dissolution into 70-μl nuclease-free water. In the verification phase of methylation markers, we use DNA pools for reducing the expense of DNA’s amount, cost, and the time. It provides a rapid and cost-effective method. In the validation phase, we indeed analyzed these samples individually.

For quantifying DNA methylation levels, we used bisulfite pyrosequencing and quantitative methylation-specific PCR (qMSP) assays. All primers are listed in Additional file 1: Table S3. Bisulfite pyrosequencing primers were designed using PyroMark Assay Design 2.0 software. Sequencing amplicons were amplified in a 20-μl reaction containing 4-μl bisulfite-converted DNA, 450 nM of each primer, and 1x PyroMark Master Mix (QIAGEN). PCR was performed as follows: initial denaturation at 95 °C for 15 min, 45 cycles of 95 °C for 30 s, 60 °C for 40 s, and 72 °C for 45 s, and a final extension at 72 °C for 5 min. Sample preparation, pyrosequencing, and analysis of the results were performed using the PyroMark Q24 System (QIAGEN), according to the manufacturer’s instructions.

qMSP assays were performed as described in our previous study [42]. All biological specimens were subjected to duplicate testing for each gene using a LightCycler® 480 (Roche, Indianapolis, IN, USA). For normalizing the total input amount of DNA template in a qMSP reaction, we used the unmethylated gene *COL2A1* as a reference. DNA methylation levels were estimated using the ΔCp-value and the following formula: (Cp of Gene) − (Cp of *COL2A1*). Test results of Cp of *COL2A1* > 36 were defined as the absence of template DNA.

### Statistical analysis

The Mann–Whitney nonparametric *U* test and Kruskal–Wallis test were used to identify differences in methylation levels between ≥ 2 categories. All significant differences were assessed using a two-tailed *t* test with *P* < 0.05. For comparing the performance of each HDM gene, we calculated the sensitivity, specificity, and AUC by “closest.topleft” method and 200 bootstrapping iterations in the pROC package. For comparing the performance of combinations of HDM genes, we calculated the probability of logistic regression model for sensitivity, specificity, and AUC analysis. To translate the research results into clinical application and awareness, a logistic regression model, with ten-fold cross-validation and 200 replications, was utilized to generate a mathematical formula to predict the risk of having OC (OC-risk score). The unbiased optimism-adjusted estimates of the concordance statistic with similar absolute errors in the relatively smaller clinical dataset were generated by this method [43]. The formula was ε $+\sum \limits_{i=1}^n{\beta}_i\times {\Delta Cp}_i$; when an assessment of the genetic combination, *i* = *i*th *gene*, and ε is a variable with a value expected to be zero. For calculating ultimate estimator of the regression coefficients, ε, and *β*_*i*_, we repeated 200-times of 10-fold cross-validation, and analyzed the mean and median of all coefficients, sensitivity, specificity, and AUC. The aforementioned analyses and plots were performed using the statistical package in R (version 3.3.2) or MedCalc version 18 (MedCalc Software bvba, Ostend, Belgium; http://www.medcalc.org; 2018).

## Supplementary information

**Additional file 1.** **Figure S1.** The differential methylation analysis on three datasets. **Figure S2.** The verification of HIST1H3E DNA methylation using bisulfite pyrosequencing in ovarian tissues **Table S1.** Clinicopatological features of clinical samplings for identification of DNA methylomics profiles **Table S2.** Summary of KEGG and Reactome pathways related to 151 differential methylation of candidate genes in ovarian cancer **Table S3.** The primers for quantitative methylation-specific PCR and bisulfite pyrosequencing **Table S4.** Summary methylation level of 151 DM genes in TMU-tissue set **Table S5.** Summary of differential methylation levels in eight genes from DNA pools of cervical scrapings **Table S6.** Comparisons of the methylation level between young and old cases using normal cervical scrapings.

## Data Availability

The datasets used and analyzed during the current study are available where appropriate from the corresponding author, upon reasonable request.

## Abbreviations

AMP: Adenosine monophosphate
AOCS: Australian Ovarian Cancer Study
*AOX1*: Aldehyde oxidase 1
AUCs: Area under the receiver operating characteristic curves
CA-125: Cancer antigen 125
CC: Cervical cancer
CTCs: Circulating tumor cells
ctDNA: Circulating tumor DNA
DAVID: Database for Annotation, Visualization and Integrated Discovery
ECs: Endometrial cancers
GEO: Gene Expression Omnibus
HDM: Highly differentially methylated
IMP: Inosine monophosphate
KEGG: Kyoto Encyclopedia of Genes and Genomes
MBD2: Methyl-CpG-binding domain protein 2
MeV: Multiple Experiment Viewer
*NRN1*: Neuritin 1
OC: Ovarian cancer
OS: Overall survival
Pap: Papanicolaou
PLCO: Prostate, Lung, Colorectal, and Ovarian
qMSP: Quantitative methylation-specific polymerase chain reaction
SNP: Single nucleotide polymorphism
*TBX15*: T-box-15
TMU-A: Taipei Medical University-A
TSS: Transcriptional start site
TVU: Transvaginal ultrasound
UKCTOCS: UK Collaborative Trial of Ovarian Cancer Screening
ΔCp: Difference of crossing points
