# Supplementary material for: Ovarian cancer detection by DNA methylation in cervical scrapings
Source: Clin Epigenetics. 2019 Nov 27;11:166. doi: 10.1186/s13148-019-0773-3 (PMC6881994; doi:10.1186/s13148-019-0773-3)
Supplement: Supplementary file 1 — Additional file 1. Figure S1. The differential methylation analysis on three datasets. Figure S2. The verification of HIST1H3E DNA methylation using bisulfite pyrosequencing in ovarian tissues Table S1. Clinicopatological features of clinical samplings for identification of DNA methylomics profiles Table S2. Summary of KEGG and Reactome pathways related to 151 differential methylation of candidate genes in ovarian cancer Table S3. The primers for quantitative methylation-specific PCR and bisulfite pyrosequencing Table S4. Summary methylation level of 151 DM genes in TMU-tissue set Table S5. Summary of differential methylation levels in eight genes from DNA pools of cervical scrapings Table S6. Comparisons of the methylation level between young and old cases using normal cervical scrapings. [file 13148_2019_773_MOESM1_ESM.pdf]

Additional file

DNA Methylation for Ovarian Cancer Detection by Cervical Scrapings

Tzu-I Wu[1,2], Rui-Lan Huang[1,3], Po-Hsuan Su[4], Shih-Peng Mao[3], Chen-Hsuan Wu[5,6] and Hung-Cheng Lai[1,3,4,7]

[1]Department of Obstetrics and Gynecology, School of Medicine, College of Medicine, Taipei Medical University, Taipei, Taiwan
[2]Department of Obstetrics and Gynecology, Wan Fang Hospital, Taipei Medical University, Taipei, Taiwan
[3]Department of Obstetrics and Gynecology, Shuang Ho Hospital, Taipei Medical University, New Taipei, Taiwan
[4]Translational epigenetic Center, Shuang Ho Hospital, Taipei Medical University, New Taipei, Taiwan
[5]Graduate Institute of Clinical Medical Sciences, Chang Gung University College of Medicine, Tao-Yuan, Taiwan
[6]Department of Obstetrics and Gynecology, Kaohsiung Chang Gung Memorial Hospital and Chang Gung University College of Medicine, Kaohsiung, Taiwan.
[7]Department and Graduate Institute of Biochemistry, National Defense Medical Center, Taipei, Taiwan

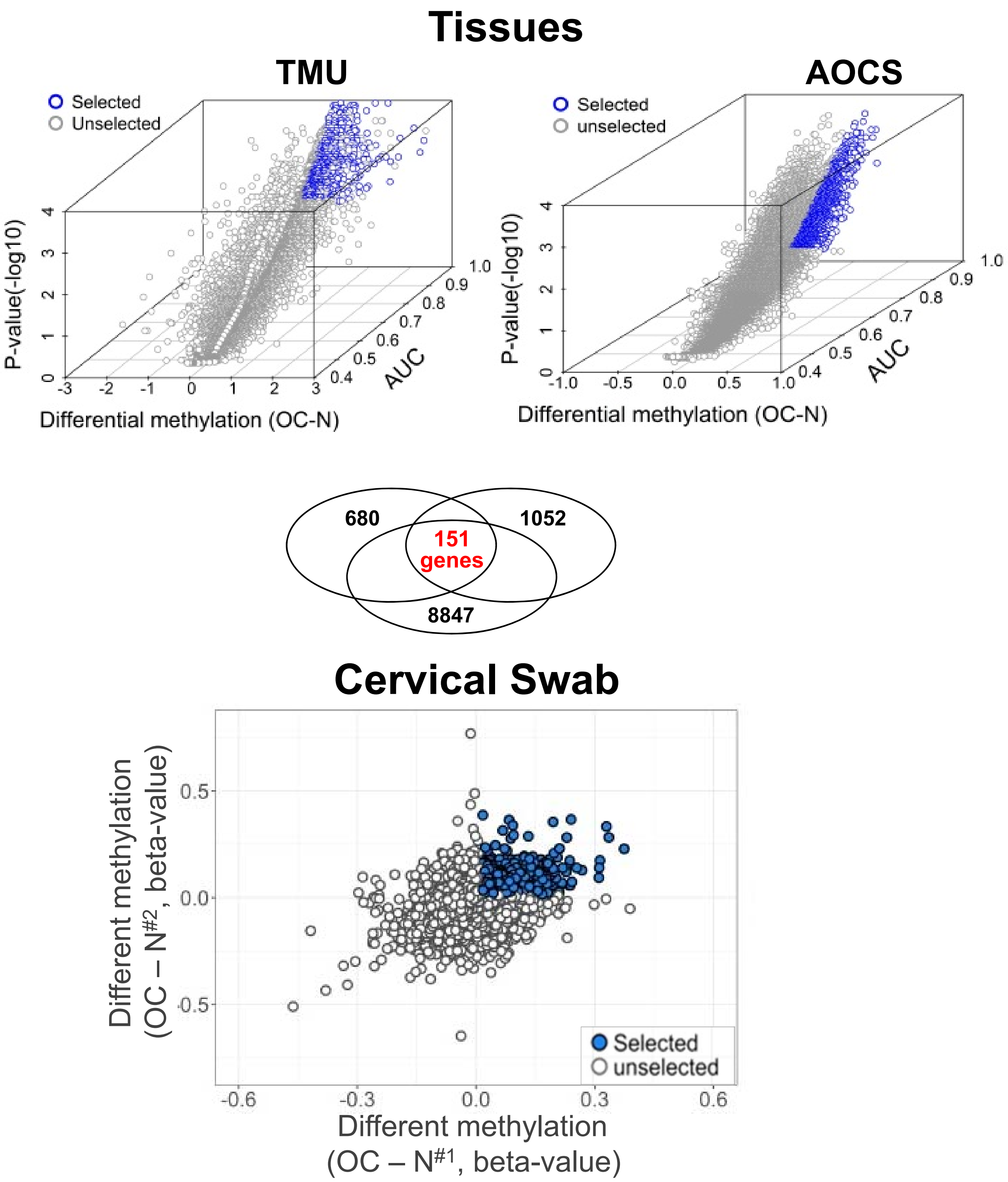

Additional file. Figure S1. The differential methylation analysis on three datasets

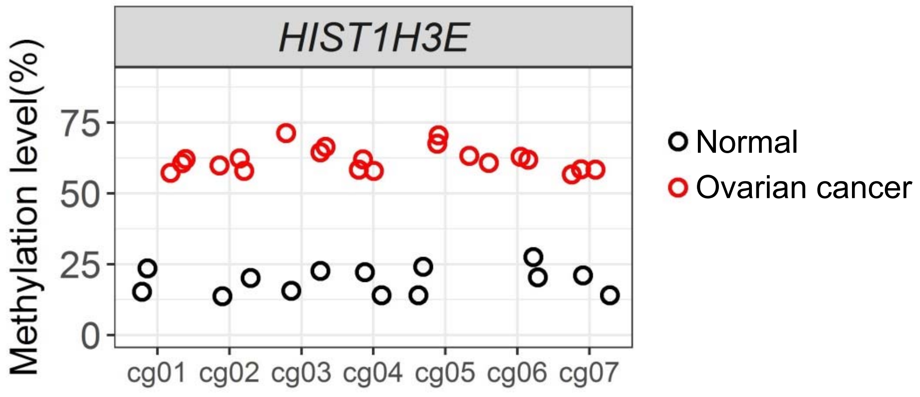

Additional file. Figure S2. The verification of HIST1H3E DNA methylation using bisulfite pyrosequencing in ovarian tissues.
We tested the methylation levels by using 14 normal controls and 21 ovarian cancer tissues.

Additional File. Table S1

Clinicopatological features of clinical samplings for identification of DNA methylomics profiles

	TMU-A (tissue)			AOCS (tissue)			TMU-B (cervcial scrapings)		
	Serous type OC		Normal	Serous type OC[a]		Normal	Serous type OC		Normal
	Number (%)		Number	Number (%)		Number	Number (%)		Number
Total number of cases	50		6	79		6	5		10
Age (years)									
Mean ± SD	58.1 ± 12.1		51.3 ± 16.4	60.1 ± 8.7		NA	65.8 ± 14.0		40.9 ± 4.8
FIGO stage									
Stage 1	5	(10.0)		0	(0.0)		0	(0.0)	
Stage 2	6	(12.0)		0	(0.0)		2	(40.0)	
Stage 3	35	(70.0)		67	(84.8)		1	(20.0)	
Stage 4	4	(8.0)		12	(15.2)		2	(40.0)	
Grading									
G1	6	(12.0)		0	(0.0)		1	(20.0)	
G2	12	(24.0)		13	(16.5)		1	(20.0)	
G3	32	(64.0)		66	(83.5)		3	(60.0)	

Abbreviations: TMU, our methylomics dataset. AOCS, the Australian Ovarian Cancer Study. OC, ovarian carcinoma. SD, Standard deviation; FIGO stage, it followed the International Federation of Gynecology and Obstetrics staging system to identify the stage. NA, none available. [a]We only used the primay tumors in the dataset (GSE65820) and downloaded from Gene Expression Omnibus.

Additional file. Table S2

Summary of KEGG and Reactome pathways related to 151 differential methylation of candidate genes in ovarian cancer

Pathway ID[a]	Category	*P*-Value	Gene Name
Total 151 DM genes			
hsa04950	Maturity onset diabetes of the young	0.01	PAX6, NEUROD1, NR5A2
R-HSA-375276	Peptide ligand-binding receptors	0.02	PRLHR, SSTR3, TRH, GHSR
hsa04915	Estrogen signaling pathway	0.03	GABBR1, PRKACA, SHC1, KCNJ3
hsa05034	Alcoholism	0.03	BDNF, SLC6A3, SHC1, HIST1H3E, HIST1H4I
hsa05030	Cocaine addiction	0.04	BDNF, SLC6A3, PRKACA
hsa04024	cAMP signaling pathway	0.04	BDNF, GABBR1, PRKACA, HHIP, GHSR
hsa04270	Vascular smooth muscle contraction	0.05	CALD1, MRVI1, ADRA1A, PRKACA
R-HSA-1442490	Collagen degradation	0.05	COL9A1, CTSK, COL14A1
R-HSA-3214841	PKMTs methylate histone lysines	0.05	HIST1H3E, HIST1H4I, NSD1
Cluster 1			
R-HSA-2129379	Molecules associated with elastic fibres	0.05	FBN2, EMILIN1
Cluster 2			
hsa04950	Maturity onset diabetes of the young	0.01	PAX6, NEUROD1, NR5A2
Cluster 3			
R-HSA-375276<br>R-HSA-416476	Peptide ligand-binding receptors<br>G alpha (q) signalling events	0.02<br>0.04	TRH, GHSR
Cluster 4			
R-HSA-452723	Transcriptional regulation of pluripotent stem cells	0.01	ZSCAN10, NR5A1

[a]The pathway enrichment analysis of differential-methylated (DM) genes used KEGG and REACTOME database. KEGG, Kyoto encyclopedia of genes and genomes; PKMT, protein lysine methyltransferase P-values calculated by modified Fisher Exact on DAVID webtool.

Additional file. Table S3

The primers for quantitative methylation-specific PCR and bisulfite pyrosequencing

Primer Name	Forward Primer Sequence (5' - 3')	Reverse Primer Sequence (5' - 3')	Sequencing primer	PCR Size (bp)
**For qMSP**				
AMPD3	TGGGTAGTTAAAGTTTAGTAGTAGTTC	ACCTCCGCATATCCAATCGT		124
AOX1	TAGATTCGGGATAAGATCGTAGAGAC	AACTTCTAAAACGACAAAACCCGC		187
CPEB1	GTGCGTTGTAGAGGGGGTCG	GCCTACCCTAAAACCCTCGC		175
MEGF11	GAGGGCGTTTGAGGTTC	CTAACTTAACCTCACTAAAAATATCGT		151
NRN1	GGGGCGGGAGCGTTTTTGTTAC	CGCGCGCAACCTCCCGA		172
PCDHGA11	TAAGAATGGGTTTTAGGCGTCGT	AACTCTAAACCGATATTCCGCGAT		256
PHOX2A	GAGGGAGGTATGGAGATAGAAGGGGTC	CGCTCTCAACCTCTACTCCCGC		187
TBX15	GGTTAAGTAGGTTAGTCGTGACGA	TCCGCAATCCGACAAAAAATTCG		200
**For Bisulfite Pyrosequencing PCR**				
HIST1H3E	GTTTTTGAGAGTTGTTTTAGTGGTTAGT	#ACCAATCTTCCTAACTCATTTACTT	GTTGTTTTAGTGGTTAGTT	124

The 5'-end biotinylated primer, All annealing temperatures are 60°C

Additional file. Table S4. Summary methylation level of 151 DM genes in TMU-tissue set

Gene Name	Chr	Strand	Start	End	RefSeq	The average of mapped reads		
						SeOC	Normal	SeOC-Normal
ACOT11	1	+	54785394	54785393	NM_015547	0.84	0.18	0.66
ADAM33	20	-	3609778	3609777	NM_001282447	1.10	0.25	0.85
ADAMTSL4	1	+	148787468	148787467	NM_019032	0.38	0.09	0.29
ADRA1A	8	-	26777839	26777838	NM_000680	0.92	0.17	0.75
AKNA	9	-	116195506	116195505	NM_030767	0.61	0.24	0.37
AMIGO3	3	-	49731242	49731241	NM_198722	0.68	0.24	0.44
AMPD3	11	+	10433056	10433055	NM_001025390	0.43	0.06	0.37
ANKRD53	2	+	71058082	71058081	NM_024933	0.56	0.05	0.51
AOX1	2	+	201157975	201157974	NM_001159	1.35	0.30	1.05
ARHGAP27	17	-	40862446	40862445	NM_001282290	1.68	0.45	1.23
ASB16	17	+	39602599	39602598	NM_080863	0.41	0.15	0.26
BARHL2	1	-	90954382	90954381	NM_020063	0.98	0.04	0.94
BDNF	11	-	27699181	27699180	NM_170731	0.45	0.03	0.43
BNC1	15	-	81743472	81743471	NM_001717	0.70	0.18	0.52
BZRAP1	17	-	53760151	53760150	NM_001261835	0.83	0.16	0.67
C1orf86	1	-	2128032	2128031	NM_001146310	0.97	0.23	0.74
C1QL4	12	-	48016238	48016237	NM_001008223	0.87	0.36	0.50
CACNA1E	1	+	179718308	179718307	NM_001205293	0.67	0.04	0.63
CACYBP	1	+	173234193	173234192	NM_001007214	1.04	0.19	0.86
CADPS	3	-	62835104	62835103	NM_003716	0.50	0.05	0.45
CALD1	7	+	134225690	134225689	NM_033139	0.45	0.23	0.21
CBLN4	20	-	54012935	54012934	NM_080617	1.23	0.14	1.09
CCDC140	2	+	222870109	222870108	NM_153038	1.02	0.13	0.90
CDO1	5	-	115179304	115179303	NM_001801	1.24	0.22	1.02
CHAT	10	+	50486146	50486145	NM_020984	0.16	0.01	0.15
CIDEB	14	-	23849416	23849415	NM_014430	0.71	0.18	0.54
COL14A1	8	+	121205527	121205526	NM_021110	0.76	0.06	0.70
COL9A1	6	-	71048632	71048631	NM_078485	1.52	0.16	1.36
COLEC11	2	+	3619296	3619295	NM_024027	2.46	0.92	1.54
CPEB1	15	-	81112822	81112821	NM_030594	1.29	0.28	1.01
CRYGD	2	-	208696558	208696557	NM_006891	1.35	0.40	0.94
CTSK	1	-	149046541	149046540	NM_000396	0.70	0.13	0.57
CYB5R3	22	-	41369584	41369583	NM_001171660	0.50	0.21	0.29
CYP1B1	2	-	38155827	38155826	NM_000104	0.36	0.04	0.33
CYP26C1	10	+	94810010	94810009	NM_183374	2.66	0.49	2.17
DAPK1	9	+	89302269	89302268	NM_001288729	0.27	0.02	0.25
DBX1	11	-	20137446	20137445	NM_001029865	0.86	0.04	0.83
DLX4	17	+	45404128	45404127	NM_001934	0.43	0.09	0.35
DMRT3	9	+	965967	965966	NM_021240	0.36	0.09	0.27
DMRTA2	1	-	50660707	50660706	NM_032110	1.08	0.21	0.87
DNHD1	11	+	6474101	6474100	NM_144666	2.12	0.93	1.19
DRGX	10	-	50273068	50273067	NM_001276451	0.43	0.01	0.41
ELAVL4	1	+	50285272	50285271	NM_001144777	0.62	0.19	0.43
ELTD1	1	-	79244083	79244082	NM_022159	0.70	0.23	0.47

Additional file. Table S4. Summary methylation level of 151 DM genes in TMU-tissue set

Gene Name	Chr	Strand	Start	End	RefSeq	The average of mapped reads		
						SeOC	Normal	SeOC-Normal
EMILIN1	2	+	27153938	27153937	NM_007046	1.16	0.14	1.02
ENG	9	-	129648267	129648266	NM_001278138	1.13	0.37	0.76
ENG	9	-	129655873	129655872	NM_000118	0.78	0.22	0.56
EOMES	3	-	27738210	27738209	NM_001278183	0.29	0.01	0.27
EVX1	7	+	27247688	27247687	NM_001989	0.86	0.10	0.76
FAIM2	12	-	48583027	48583026	NM_012306	1.21	0.22	0.99
FAM115A	7	-	143212399	143212398	NM_001206941	1.34	0.09	1.25
FBN2	5	-	127900634	127900633	NM_001999	0.23	0.00	0.23
FER1L5	2	+	96671200	96671199	NM_001293083	0.68	0.35	0.33
FEZF1	7	-	121730801	121730800	NM_001160264	0.29	0.06	0.24
FHL2	2	-	105381113	105381112	NM_201555	1.55	1.20	0.35
FOXB2	9	+	78823390	78823389	NM_001013735	1.48	0.07	1.41
FOXG1	14	+	28305028	28305027	NM_005249	0.41	0.06	0.35
GABBR1	6	-	29702984	29702983	NM_021903	0.22	0.00	0.22
GATA3	10	+	8135672	8135671	NM_002051	0.50	0.03	0.47
GHSR	3	-	173647940	173647939	NM_004122	2.47	0.15	2.32
GSX1	13	+	27263779	27263778	NM_145657	0.69	0.07	0.62
HHIP	4	+	145785597	145785596	NM_022475	0.76	0.18	0.58
HIST1H3E	6	+	26332361	26332360	NM_003532	3.54	0.88	2.66
HIST1H4I	6	+	27214066	27214065	NM_003495	0.97	0.09	0.88
HNRNPF	10	-	43211285	43211284	NM_001098208	0.42	0.04	0.38
HOXA9	7	-	27170674	27170673	NM_152739	1.88	0.20	1.68
HOXC12	12	+	52633980	52633979	NM_173860	0.56	0.06	0.51
HOXD12	2	+	176671775	176671774	NM_021193	1.99	0.23	1.76
IFFO1	12	-	6534510	6534509	NM_080730	1.79	0.16	1.64
IRX2	5	-	2803769	2803768	NM_033267	0.70	0.03	0.66
KCNJ3	2	+	155262338	155262337	NM_002239	1.10	0.19	0.91
KCTD17	22	+	35776721	35776720	NM_024681	0.68	0.14	0.55
KIFC3	16	-	56392940	56392939	NM_001130100	0.34	0.11	0.23
KLHL33	14	-	19972641	19972640	NM_001109997	1.89	0.64	1.25
L1TD1	1	+	62432061	62432060	NM_001164835	1.31	0.41	0.90
LFNG	7	+	2523022	2523021	NM_002304	0.45	0.22	0.23
LHX6	9	-	124022840	124022839	NM_001242335	0.72	0.09	0.62
LHX8	1	+	75372154	75372153	NM_001256114	0.50	0.07	0.43
LIMA1	12	-	48901755	48901754	NM_001113547	0.46	0.21	0.25
LIMD2	17	-	59130251	59130250	NM_030576	0.35	0.00	0.35
LRRFIP1	2	+	238199962	238199961	NM_001137550	0.56	0.15	0.41
LYNX1	8	-	143854746	143854745	NM_177477	1.11	0.30	0.81
MAEA	4	+	1292578	1292577	NM_001297433	1.70	0.76	0.94
MEGF11	15	-	64332129	64332128	NM_032445	0.54	0.00	0.54
MKX	10	-	28073784	28073783	NM_173576	0.49	0.01	0.48
MOS	8	-	57188095	57188094	NM_005372	0.83	0.09	0.74
MRVI1	11	-	10670697	10670696	NM_001100167	0.64	0.12	0.52
NACAD	7	-	45094018	45094017	NM_001146334	0.52	0.10	0.42

Additional file. Table S4. Summary methylation level of 151 DM genes in TMU-tissue set

Gene Name	Chr	Strand	Start	End	RefSeq	The average of mapped reads		
						SeOC	Normal	SeOC-Normal
NEFM	8	+	24827359	24827358	NM_001105541	1.19	0.10	1.09
NETO1	18	-	68684790	68684789	NM_138966	0.69	0.06	0.63
NEUROD1	2	-	182252637	182252636	NM_002500	0.52	0.05	0.47
NGEF	2	-	233500105	233500104	NM_001114090	0.99	0.30	0.70
NKX2-4	20	-	21325047	21325046	NM_033176	0.77	0.05	0.72
NKX2-6	8	-	23619056	23619055	NM_001136271	1.30	0.16	1.14
NKX3-2	4	-	13154212	13154211	NM_001189	0.59	0.08	0.51
NR5A1	9	-	126308520	126308519	NM_004959	1.75	0.41	1.34
NR5A2	1	+	198277575	198277574	NM_001276464	0.79	0.08	0.70
NRN1	6	-	5948276	5948275	NM_001278711	1.61	0.24	1.38
NSD1	5	+	176491685	176491684	NM_172349	0.45	0.15	0.30
OTX2	14	-	56345947	56345946	NM_001270524	1.63	0.10	1.52
OVOL1	11	+	65310080	65310079	NM_004561	0.61	0.17	0.44
PAX6	11	-	31795085	31795084	NM_001127612	0.68	0.07	0.61
PCDHA9	5	+	140206540	140206539	NM_031857	0.62	0.21	0.41
PCDHB4	5	+	140480515	140480514	NM_018938	1.27	0.45	0.82
PCDHGA11	5	+	140779720	140779719	NM_032092	2.62	0.45	2.17
PCDHGA3	5	+	140702784	140702783	NM_018916	1.54	0.73	0.81
PCDHGA5	5	+	140723081	140723080	NM_018918	2.60	0.94	1.66
PCDHGB2	5	+	140718886	140718885	NM_018923	1.65	0.86	0.78
PCDHGB6	5	+	140766953	140766952	NM_018926	1.06	0.27	0.79
PCDHGB7	5	+	140776443	140776442	NM_018927	1.02	0.24	0.78
PFN3	5	-	176759243	176759242	NM_001029886	1.92	0.40	1.51
PHF19	9	-	122695995	122695994	NM_001286840	0.78	0.23	0.55
PHOX2A	11	-	71631868	71631867	NM_005169	1.46	0.29	1.17
PHYHD1	9	+	130721994	130721993	NM_001100876	0.93	0.25	0.69
PPP1R17	7	+	31692155	31692154	NM_001145123	0.37	0.06	0.32
PRKACA	19	-	14084992	14084991	NM_207518	0.79	0.12	0.67
PRLHR	10	-	120344150	120344149	NM_004248	1.42	0.33	1.09
RAB3IL1	11	-	61443317	61443316	NM_001271686	0.80	0.40	0.39
RAX	18	-	55090605	55090604	NM_013435	0.88	0.03	0.85
RCAN2	6	-	46400588	46400587	NM_005822	0.47	0.12	0.35
RUNX3	1	-	25128357	25128356	NM_004350	1.06	0.10	0.96
SATB2	2	-	200037076	200037075	NM_001172517	0.64	0.10	0.54
SGCD	5	+	155685344	155685343	NM_000337	0.33	0.11	0.21
SHC1	1	-	153208847	153208846	NM_183001	0.43	0.04	0.39
SIX3	2	+	45021540	45021539	NM_005413	0.56	0.07	0.49
SKOR2	18	-	43028552	43028551	NM_001037802	1.13	0.19	0.94
SLC2A14	12	-	7915902	7915901	NM_001286236	1.98	1.16	0.82
SLC6A3	5	-	1497543	1497542	NM_001044	0.89	0.17	0.72
SLC7A14	3	-	171785557	171785556	NM_020949	0.97	0.24	0.73
SMAD3	15	+	65244546	65244545	NM_001145104	2.46	0.59	1.87
SOX1	13	+	111768913	111768912	NM_005986	0.57	0.03	0.54
SPAG6	10	+	22673379	22673378	NM_012443	1.08	0.13	0.95

Additional file. Table S4. Summary methylation level of 151 DM genes in TMU-tissue set

Gene Name	Chr	Strand	Start	End	RefSeq	The average of mapped reads		
						SeOC	Normal	SeOC-Normal
SPATS2L	2	+	200877848	200877847	NM_001282743	0.70	0.19	0.50
SSH1	12	-	107744456	107744455	NM_001161331	2.17	1.05	1.12
SSTR3	22	-	35937362	35937361	NM_001051	1.07	0.29	0.78
ST8SIA3	18	+	53169718	53169717	NM_015879	0.27	0.00	0.27
SYNGR1	22	+	38089120	38089119	NM_145738	0.67	0.14	0.53
TBX15	1	-	119332702	119332701	NM_152380	1.79	0.27	1.52
TBX20	7	-	35259236	35259235	NM_001166220	1.20	0.16	1.04
TBX5	12	-	113329630	113329629	NM_000192	1.14	0.21	0.93
TCAP	17	+	35074124	35074123	NM_003673	0.49	0.19	0.30
TCF21	6	+	134250952	134250951	NM_003206	0.94	0.10	0.84
TGFB1I1	16	+	31391026	31391025	NM_015927	0.88	0.34	0.54
TMEM220	17	-	10573371	10573370	NM_001004313	0.38	0.11	0.27
TRH	3	+	131174925	131174924	NM_007117	1.82	0.52	1.30
VAMP5	2	+	85664041	85664040	NM_006634	0.89	0.07	0.82
ZBTB16	11	+	113434640	113434639	NM_006006	0.62	0.03	0.60
ZIC1	3	+	148608870	148608869	NM_003412	2.18	0.16	2.02
ZIC4	3	-	148604997	148604996	NM_001168379	0.67	0.07	0.60
ZIC5	13	-	99421179	99421178	NM_033132	0.67	0.14	0.53
ZSCAN10	16	-	3081862	3081861	NM_032805	1.79	0.92	0.87
CLIP4	2	+	29190795	29190794	NM_001287527	0.43	0.15	0.28

Chr, chromosome; RefSeq, the Reference Sequence; SeOC, serous ovarian cancer; DM, differential methylation. The mRNA locations indicated by hg18 database.

Supplementary Table S5. Summary of differential methylation levels in eight genes from DNA pools of cervical scrapings

Gene Name	ΔCp of Normal (Mean±SE)	ΔCp of OC (Mean±SE)	ΔΔCp (N-OC)	Clustering subgroup
AOX1	10.1 ± (0.47)	3 ± (0.34)	7.1	1
CPEB1	4.7 ± (0.49)	1.8 ± (0.07)	2.9	1
PHOX2A	2.6 ± (0.76)	-0.2 ± (0.18)	2.8	1
MEGF11	6.2 ± (0.17)	4.5 ± (0.39)	1.7	2
AMPD3	4.9 ± (0.02)	3.4 ± (0.14)	1.5	2
TBX15	12.5 ± (1.93)	6 ± (1.23)	6.5	3
NRN1	9.6 ± (0.93)	3.5 ± (0.42)	6.1	3
PCDHGA11	15.3 ± (0.53)	9.7 ± (1.60)	5.6	3

SE: standard error; OC, ovairan cancer

Table S6 . Comparisons of the methylation level between young and old cases using normal cervical scrapings

Groups of age	<50 (mean ± SE)	≥ 50 (mean ± SE)	P^a
Sample size	32	20	
dCp of AMPD3	3.8 ± 0.2	3.6 ± 0.2	0.42
dCp of NRN1	3.6 ± 0.3	3.0 ± 0.5	0.31
dCp of TBX15	8.2 ± 0.4	7.2 ± 0.5	0.12
OC-risk score (AMPD3+NRN1+ TBX5)	-1.5 ± 0.3	-0.6 ± 0.5	0.31

SE, standard error. [a]*P* values calculated by two-tailed Welch-test
